# SKINREMS—A New Method for Assessment of the Niacin Skin Flush Test Response in Schizophrenia

**DOI:** 10.3390/jcm9061848

**Published:** 2020-06-13

**Authors:** Hanna Karakula-Juchnowicz, Joanna Rog, Piotr Wolszczak, Kamil Jonak, Ewa Stelmach, Paweł Krukow

**Affiliations:** 1 1st Department of Psychiatry, Psychotherapy and Early Intervention, Medical University of Lublin, 20-439 Lublin, Poland; hanna.karakula-juchnowicz@umlub.pl; 2 Department of Clinical Neuropsychiatry, Medical University of Lublin, 20-439 Lublin, Poland; k.jonak@pollub.pl (K.J.); pawelkrukow@umlub.pl (P.K.); 3 Department of Automation, Faculty of Mechanical Engineering, Lublin University of Technology, 20-618 Lublin, Poland; p.wolszczak@pollub.pl; 4 Department of Biomedical Engineering, Lublin University of Technology, 20-618 Lublin, Poland; 5 2nd Department of Psychiatry and Psychiatric Rehabilitation, Medical University of Lublin, 20-439 Lublin, Poland; ewastelmach@umlub.pl

**Keywords:** schizophrenia, bipolar disorder, biomarker, niacin, essential fatty acids

## Abstract

Schizophrenia has been considered a disorder linked with faulty lipid homeostasis, and the proposed tool for assessment of these disruptions is the niacin skin flush test. The aims of the study were: 1. Create a new tool to analyze results of the niacin skin flush test more precisely and objectively. 2. Verify the utility of a self-created tool for differentiating between schizophrenia (SZ; *n* = 56), bipolar disorder (BD; *n* = 29) and healthy control (HC; *n* = 45) individuals. The proposed developed method, based on the Skin Reaction Measurement Computer System (SKINREMS), allows one to evaluate the response to the niacin skin flush test quickly and objectively. SKINREMS showed good accuracy in discriminating SZ from BD (with sensitivity 91% and specificity 72%), and SZ from HC (71% and 66%, respectively), and sufficient but not excellent accuracy in discriminating BD from HC (55% and 54%, respectively). The pathophysiological pathways and features shared by schizophrenia and bipolar disorder may be the reason for difficulties in fully discriminating between these two mental disorders using the niacin challenge test. The management of disruptions in the phospholipid metabolism and the inflammatory process could potentially become an individualized form of therapy in a subgroup of psychiatric patients.

## 1. Introduction

Schizophrenia is a chronic mental disorder affecting more than 21 million people worldwide [1]. The severity of its psychopathological symptoms, including changes in thinking, perception, behavior, personality and functioning, makes schizophrenia a complex disorder, leading to low quality of life and disability [2]. The pathogenesis of the illness remains unclear; however, the number of studies confirming the influence of both genetic and environmental factors is still growing [1,2].

Many theories have been proposed over the years to explain the pathogenesis of schizophrenia. Among them, there is the membrane hypothesis of schizophrenia, formulated by David Horrobin, with a large body of evidence supporting its validity [3]. Horrobin had concentrated his research on the biochemistry of lipids and their metabolism [4]. Phospholipase A2 (PLA-2) is an enzyme engaged in releasing fatty acids from the phospholipids of cell membranes (mainly arachidonic acid, AA). Excessive activation of PLA-2 leads to the synthesis of prostaglandins via cyclooxygenase 1 and 2 (COX-1, COX-2), causing the cascade of inflammatory reactions [5]. The consequences of biochemical pathway-disruption are the deficiency of fatty acids, defects in prostaglandin transformations, and the abnormal structure of phospholipids cell membranes [3,5].

Horrobin’s theory is supported by post-mortem observational studies, which confirmed reduced levels of AA in patients with schizophrenia [6,7]. More and more studies indicate that the niacin test may be a simple, quick and non-invasive method to reveal disruptions in the metabolic pathway of fats [8,9,10,11,12]. A topical application of niacin to skin activates the G-coupled nicotinic acid receptors, which induces the activation of phospholipase A2 (PLA-2), the release of fatty acids (mainly AA, but also eicosapentaenoic acid—EPA) from cell membranes, and (via a mechanism mediated by COX-1 and COX-2) the formulation of prostaglandins (E2 and D2). Niacin priming can increase the serum concentration of prostaglandins up to several hundred times [13,14]. Prostaglandins stimulate the production of cyclic AMP, which causes the relaxation of smooth muscles in skin capillary walls, and consequent vasodilatation. The metabolic pathway affects topical flow of blood and, in consequence, results in skin flushing. A great number of chemical compounds (e.g., prostacyclins, thromboxanes) that are important for the proper functioning of the central nervous system (CSN) are also involved in mechanisms contributing to skin oedema [5]. The substantial body of evidence supports observations of impaired reactions to niacin in individuals with schizophrenia. Some of the studies suggest that a blunted skin flush response satisfies the criteria for a schizophrenia endophenotype [8], fulfilling all five criteria proposed in 2003 by Gottesman and Goul [15], and may be considered as a potential biomarker of schizophrenia [16].

In addition, an abnormal response to aqueous methyl nicotinate (AMN) shows linkage to two genes at human chromosome 14q32.12, related to susceptibility to, or the clinical features of, schizophrenia [9].

According to clinical staging, the response to the niacin skin flush test is a specific marker for all stages of schizophrenia [17,18]. Nevertheless, depending on the method of niacin challenge and the criteria for defining abnormality of response, the niacin-blunted subgroup in SZ was estimated to range from 24% to 90% of the examined subjects (with the average of 70% [8]), compared to 0–64% in the healthy controls [5].

The inconsistent results may be caused by the lack of an objective and precise tool for assessing responses to niacin challenge [5]. In 2010, Nadalin et al. published a review of methods used to perform and evaluate the niacin test. Most of the papers were focused on subjective assessment by visual inspection. Some authors tried to find more objective methods, e.g., Doppler Flowmetry, optical reflection spectroscopy (ORS) or analysis with Photoshop program [5]. The variety of methods used by researchers makes it difficult to compare the results of different studies, and may lead to discrepancies in the number of individuals with diminished or no response. These considerations became a starting point for developing a new tool for niacin response assessment.

The aims of the study were to create a new, more precise and quick method for the objective assessment of niacin challenge response, and to verify the utility of a tool, developed by our research team, to differentiate (1) patients suffering from schizophrenia and healthy individuals, (2) bipolar disorder patients and healthy individuals, and (3) schizophrenia patients and bipolar disorder patients.

## 2. Materials and Methods

### 2.1. Participants

The study included in- and out-patients: 56 patients diagnosed with schizophrenia (SZ group) and 29 patients diagnosed with bipolar disorder (BD group), and 45 healthy individuals as a control group (HC group).

The inclusion criteria for patients were diagnosis of schizophrenia (SZ group), diagnosis of bipolar disorder (BD group) based on DSM-5 criteria, age between 16 and 65 years, and ability to provide written informed consent as determined by clinical examination and verbal communication.

Patients were excluded from the study if they had somatic diseases which in the investigators’ opinion may affect the results of the study (allergy, asthma, skin diseases, cardiac diseases, cancers, autoimmune diseases, dermatological lesions), suffered from substance use disorders (except nicotine and caffeine), or if they, within the previous 14 days, had not taken drugs or dietary supplements affecting the metabolism of prostaglandins (anti-inflammatory drugs, glucocorticosteroids, fatty acids supplements). Pregnant and breast-feeding women were also excluded. The control group consisted of volunteers aged 16–65. The exclusion criteria for the control group were the same as those for the patients. Individuals with current and/or past diagnoses of psychiatric disorders, and family history of psychiatric disorders, were also excluded.

The study was approved by the Ethics Committee of Medical University of Lublin (number code of KE-0254/127/2016) and was conducted according to the principles described in the Declaration of Helsinki [19]. Informed consent was obtained from each participant (and their parents if patients were younger than 18 years) in the study.

### 2.2. Examined Variables

#### 2.2.1. Psychopathological Symptoms

The severity of psychiatric symptoms was assessed by a trained physician (E.S) with the use of Positive and Negative Syndrome Scale (PANSS) [20] for SZ group, and Young Mania Rating Scale (YMRS) [21] and Montgomery–Åsberg Depression Rating Scale (MADRS) [22] for BD group.

#### 2.2.2. Niacin Skin Flush Test

The niacin skin flush challenge test was performed as previously described in the review by Nadalin et al. [5]. We prepared Methyl nicotinate (C7H7NO2, 99%, Sigma-Aldrich Chemistry GmbH, Steinheim, Germany) solutions in three concentrations: 0.1 M; 0.01 M; and 0.001 M. In order to exclude the impact of weather conditions (sunlight, temperature) on the results, the assessment was always conducted in the same room, with the exterior roller shutters down, using constant artificial light, between 9 and 10 a.m., using the same camera and stop-watch operated by the same person (J.R.). To take the photos, 13 Mpx Huawei camera (resolution: 4224 × 3136 px, type of matrix: OmniVision OV13850) without flash was used.

For each participant, 50 µL of each solution was put onto three pieces of filter paper. Then the pieces were attached to neighboring sites on the inner side of each subject’s forearm skin with the use of non-allergic plaster. After 90 s the pieces of paper were removed. We photographed the reaction at four intervals (3, 5, 10, 15 min) after the skin challenge. To avoid possible bias, the study was blinded for the researcher performing the test who was unaware to which group a given participant belonged.

#### 2.2.3. The Niacin Response Assessment

The response to the niacin application was assessed using a computer program prepared originally for this research. In brief, the photographs obtained were uploaded to the program and the next step was to outline two areas: a flushing and a non-irritated fragment of skin, in order to determine changes in the skin color and calculate the surface area of redness.

The method is based on the comparison of the results of the images depicting the area of irritated skin on the forearm and the surrounding area of non-irritated skin with digital processing. Additionally, in each photo a ruler is visible, indicating the location of the area irritated by the nicotinamide solution and ensuring the correct conversion from pixels to millimeters. The millimeter scale of the images was used to measure the surface of the reddened areas.

To determine the lack of reaction, two researchers (J.R. and E.S.) independently analyzed the photos uploaded to the SKINREMS. An absent response was defined as the lack of visible differences in neighboring irritated and non-irritated areas of skin in a minimum of 33% (4 of 12) of the measurements.

#### 2.2.4. Description of the Skin Color Model

Measurements of the skin color were made using the software program designed by the author of this study (P.W.). This program allows for viewing photos, selecting areas for analysis and automatically calculating the coefficients characterizing skin color. Due to high sensitivity of digital cameras, the results of measurements could be disturbed by changes of lighting conditions imperceptible to the human eye, and therefore differences in the color component values between neighboring irritated and non-irritated areas of skin seem to be the optimal parameter for characterizing a skin reaction measured in the doctor’s office.

**Figure 1:**
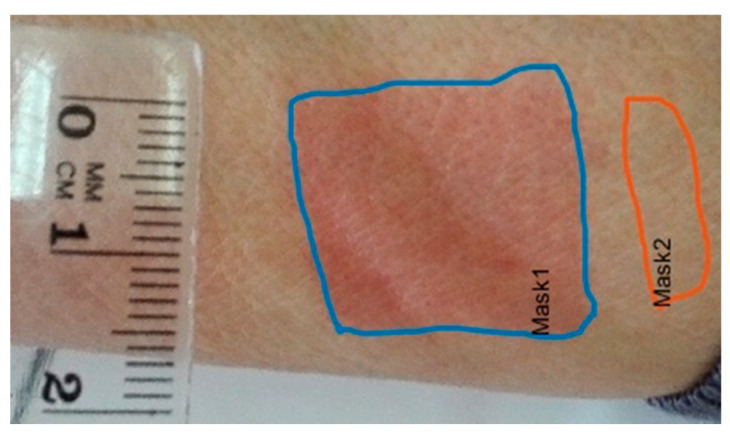
The image of the forearm of a patient from the BD group, dose 0.1 M, measurement 4.

In the pictures, two areas of skin were marked: area 1 treated with nicotinamide solution and area 2 adjacent to area 1, serving as a reference for the observation of skin color change (see Figure 1). The areas of skin were marked manually to take into account the natural variation in skin color and cases of no skin reaction. The skin reaction was characterized on the basis of differences in the color component values, expressing the color change of the irritated area. The color component values are vectors in Cartesian red, green, blue (RGB) spaces and cylindrical hue, saturation and value (HSV). Lossy conversion between RGB and HSV color models was carried out using the following equations:

(1) $$H=\{\begin{array}{c} \theta \\ 360-\theta \end{array}\;\begin{array}{c} B\leq G\\ B>G \end{array},\theta =arcos\frac{1}{2}\frac{\left(R-G\right)+\left(R-B\right)}{\sqrt{{\left(R-G\right)}^{2}+\left(R-B\right)\left(G-B\right)}}\;S=1-3\frac{\min\left(R,G,B\right)}{R+G+B}\;V=max\left(R,G,B\right)$$

The RGB and HSV spaces differ in their application. The RGB space was designed to describe the colors displayed by projectors and digital recorders. Digital displays and projectors were equipped with RGB lighting elements, and the resulting color tone was obtained by using a combination of the lamps of different intensity. Analogically, in the case of the recorders, electrical signals from three types of photosensitive elements were used. The HSV color, on the other hand, corresponds to the process of human perception. In addition to the above-presented components, the chromatic components Blue chrominance (Cb) and Red chrominance (Cr), also known as the U and V dimensions of the YUV (Y—Luminance) space used in the Phase Alternating Line (PAL) television system, were determined [23]. The conversion matrix of the RGB to YUV space is shown below (Equation (2)):

(2) $$\left[\begin{array}{c} Y\\ U\\ V \end{array}\right]=\left[\begin{array}{c} 16\\ 128\\ 128 \end{array}\right]+\left[\begin{array}{ccc} 0.257 & 0.504 & 0.098\\ -0.148 & -0.291 & 0.439\\ 0.439 & -0.368 & -0.071 \end{array}\right]\left[\begin{array}{c} R\\ G\\ B \end{array}\right]$$

The components of the YCbCr model show good results in human skin detection in a given area of an image. In the study of Surampalli et al., involving the detection of skin in digital images, the YCbCr model achieved 96.42% efficiency compared to the 3.57% efficiency of the HSV model [24]. We used the values of the conversion rates of the RGB model to the YCbCr model, presented in Equations (3) and (4):

(3) $$\left[\begin{array}{c} Y\\ Cb\\ Cr \end{array}\right]=\left[\begin{array}{ccc} 1 & 1 & 1\\ 0.148 & -0.291 & 0.439\\ 0.439 & -0.368 & -0.071 \end{array}\right]\left[\begin{array}{c} R\\ G\\ B \end{array}\right]+\left[\begin{array}{c} 16\\ 128\\ 128 \end{array}\right]$$

(4) Cb=0.148R−0.291G+0.439B+128Cr=0.439R−0.368G−0.071B+128

In this case, the 8-bit pixel values for Cr (red chrominance value) will range from 140 to 165, and the blue chrominance from 140 to 195. The authors prepared a special software to modify the threshold values Cr, Cb and Y (Hue, in range 0.01–0.1).

The areas treated with the solution were manually marked on the images using the software. The control regions were located in areas subjected to similar light exposure levels as the irritated areas, along the axis of the forearm (see Figure 1), so as to compensate for the uneven illumination of the skin of the oval forearm. The ruler was put next to the areas to determine the image scale in millimeters (mm).

Then, the color characteristics were calculated for the selected areas 1 and 2, according to the Equations (1) and (4). Those areas were processed automatically. The set of representative values included the area A of the region (1), and pairs of the following statistics: mean, median, mode, range, minimum and maximum values of components R, G, B, H, S, V, Cr and Cb (97 variables in total: 6 statistics × 8 components × 2 areas + 1 area). The measurement results were collected in the Microsof SQL Serfer database. The use of the database made it possible to prepare various combinations of variables adapted to the needs of statistical analysis.

The values referring to (1) areas of redness and (2) control areas of skin were used to calculate the relative statistics (differences between the areas). Statistics characterizing the variability were used to evaluate the correctness of the data. For selected variables, normal distribution was also assessed.

### 2.3. Statistical Analysis

Demographic and clinical variables were compared among the three groups with Kruskal–Wallis test as a non-parametric ANOVA. Further, nominal variables were compared with χ^2^ test, and if any variables were noted only in two groups (e.g., antipsychotic treatment), a non-parametric Mann–Whitney U test was applied, due to the non-Gaussian distribution of the obtained results.

Most of the studies compare the niacin skin flush test between individuals with schizophrenia and healthy controls [5,25,26]. To check the utility of our tool in differentiating between two groups and selecting factors that would provide the highest sensitivity and specificity of the test (which could be useful in performing clinical assessments of patients), first the response on the niacin solution between HC and SZ group was compared.

If the test differentiated between these two groups, we compared the reactions of patients from the two clinical settings (SZ and BD).

In this way, we wanted to confirm the hypothesis that the sensitivity and specificity of our tool were high enough to identify patients with the specific diagnosis (schizophrenia). The next step was to select components of the test differentiating BD from HC group.

The most recommended and methodologically correct analysis compares three groups, however, it could blur results of the comparison between two groups.

Therefore, we decided to perform three independent tests comparing two groups, in search of components which could help confirm our hypothesis. We also decided to compare the groups in pairs, in order to minimize the risk of Type I errors in the analysis [27].

To assess differences between the groups, we conducted a multivariate analysis of variance (MANOVA). The dependent variables included various markers of skin flush induced by niacin exposition. MANOVA was applied to verify the effect of three doses of niacin in solution (0.001 M, 0.01 M and 0.1 M) and four temporal intervals after 90 s of niacin exposure (3, 5, 10, 15 min). The final MANOVA model was 2 groups × 4 temporal intervals for each of the three niacin solutions (0.001 M, 0.01 M and 0.1 M). Partial eta squared (ƞ^2^_p_) values were calculated to assess the effect size. The initial level of statistical significance for MANOVA computations was set at *p* < 0.05, however, the final level of significance has been adjusted according to Bonferroni correction for multiple testing. In the Results section, a given outcome of MANOVA was indicated as statistically significant if it reached the threshold set at Bonferroni correction.

Next, the non-parametric Spearman R correlation coefficient was applied to establish any significant associations between sociodemographic variables and responses to AMN solution. These correlational analyses were performed in each group separately. To control for the possible impact of gender, in each group separately, males and females were compared with the application of Student’s t-test regarding these niacin response-related variables, which significantly differentiated the studied group, according to previously conducted computations with MANOVA.

To evaluate the diagnostic’s ability to identify examined conditions and distinguish three examined groups, the area under receiver-operating characteristic (ROC) analysis was performed [28]. A ROC graph is a technique for the organization and selection of classifiers based on their performance [29,30]. In medical decision-making procedure, the ROC curve has been widely used for assessment of sensitivity and specificity values of different medical test. In this study, the ROC curve, sensitivity and specificity values of different skin response variables were calculated, using the algorithm based on the “ROC curve” function created in Matlab environment [31].

## 3. Results

We examined 130 persons: 56 patients with schizophrenia (SZ group), 29 patients with bipolar disorder (BD group), and 45 healthy volunteers as a control group (HC). The clinical and demographic characteristics of the participants are shown in Table 1. A significant age difference was found between the three groups: the participants from HC group were younger compared to both SZ and BD group (*p* = 0.0015). There were no differences in the number of cigarettes smoked per day among the groups (*p* > 0.05), however, most of the smokers were in SZ group, while participants from HC group smoked the least. BD patients had higher BMI scores compared to HC group (*p* = 0.066). BD and SZ patients did not differ in BMI or the duration of illness (*p* > 0.05).

### 3.1. Comparison of Niacin Skin Flush Responses between Examined Groups

#### 3.1.1. Differences in Niacin Responses between Schizophrenia Group and Healthy Controls

**Figure 2:**
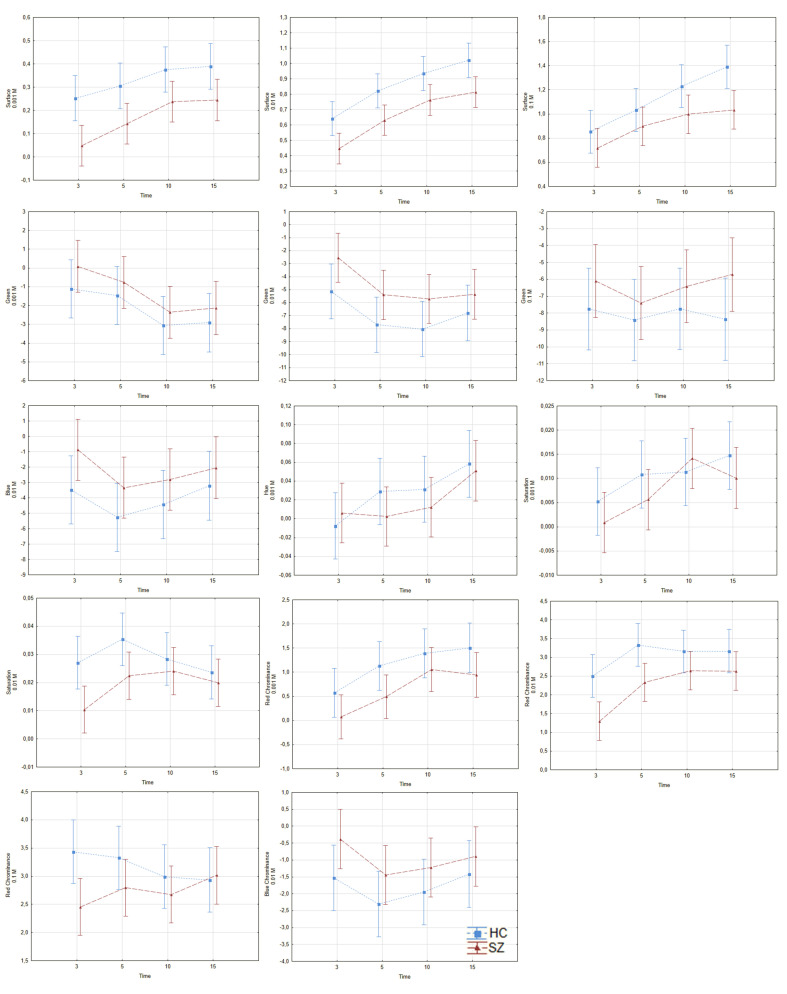
Differences in responses to niacin solution between HC and SZ. The red line—SZ group, the blue line—HC group, the vertical axis—skin color, the horizontal axis—time of measurement in minutes. Diagrams represent group and within-subjects effects described in the Section 3.1.1. SZ—schizophrenia; HC—healthy control. (**a**) surface, 0.001 M; (**b**) surface, 0.01 M; (**c**) surface, 0.1 M; (**d**) green colour, 0.001 M; (**e**) green colour, 0.01 M; (**f**) green colour, 0.1 M; (**g**) blue colour, 0.01 M; (**h**) hue, 0.001 M; (**i**) saturation, 0.001 M; (**j**) saturation, 0.01 M; (**k**) red chrominance, 0.001 M; (**l**) red chrominance, 0.01 M; (**m**) red chrominance, 0.1 M; (**n**) blue chrominance, 0.01 M.

The differences in response to the niacin between the examined groups (in pairs) are shown in Figure 2 and Supplementary Table S1 (see Supplementary Materials). After Bonferroni correction for multiple tests, we found differences in 7 of the 27 examined variables, between HC and SZ groups. The group effect was statistically significant on the surface in all examined concentrations (*p* < 0.001, ƞ^2^_p_ = 0.056; 0.06 and 0.031 for 0.001 molarity (M), 0.01 M and 0.1 M, respectively); green color was observed at 0.01 M (*p* = 0.003, ƞ^2^_p_ = 0.022); saturation at 0.01 M (*p* = 0.004, ƞ^2^_p_ = 0.021); red chrominance at 0.001 M and 0.01 M (*p* = 0.004, ƞ^2^_p_ = 0.021; and *p* < 0.001, ƞ^2^_p_ = 0.042, respectively). As it is shown in Table S1, surface and red chrominance were the components which had the highest discriminatory power and distinguished the examined groups.

The next step was to verify the hypothesis regarding a time effect. We found a time effect on the surface at all concentrations (*p* = 0.001, ƞ^2^_p_ = 0.034; *p* < 0.001, ƞ^2^_p_ = 0.124; and *p* < 0.001, ƞ^2^_p_ = 0.066, for 0.001, 0.01, and 0.1 M, respectively); green color was observed at 0.001 M (*p* = 0.007; ƞ^2^_p_ = 0.030), and red chrominance at 0.001 and 0.01 M concentrations (*p* < 0.001, ƞ^2^_p =_ 0.044; *p* < 0.001, ƞ^2^_p_ = 0.046, respectively).

#### 3.1.2. Differences in Niacin Responses between Schizophrenia Group and Bipolar Disorder Group

**Figure 3:**
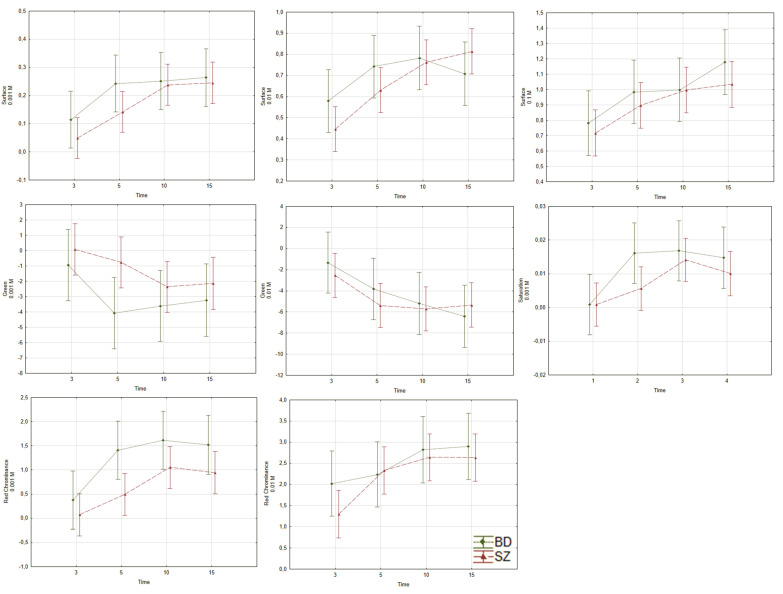
Differences in response to niacin solution between SZ and BD. The red line—SZ group, the green line—BD group, the vertical axis—skin color, the horizontal axis—time of measurement in minutes. Diagrams represent group and within-subjects effects described in Section 3.1.2. SZ—schizophrenia; BD—bipolar disorder. (**a**) surface, 0.001 M; (**b**) surface, 0.01 M; (**c**) surface, 0.1 M; (**d**) green colour, 0.001 M; (**e**) green colour, 0.01 M; (**f**) saturation, 0.001 M; (**g**) red chrominance, (**h**) 0.001 M; red chrominance, 0.01 M.

Between BD and SZ groups there was a difference in red chrominance at 0.001 M (*p* = 0.002, ƞ^2^_p_ = 0.029) with no changes in results after including the covariables (see Figure 3 and Supplementary Table S1). The time had an effect on the examined variables in all concentrations regarding surface (*p* < 0.001, ƞ^2^_p_ = 0.054; *p* < 0.001, ƞ^2^_p_ = 0.058; and *p* = 0.002, ƞ^2^_p_ = 0.045, for 0.001, 0.01 and 0.1 M, respectively), saturation at 0.001 M (*p* = 0.002, ƞ^2^_p_ = 0.044), and chrominance at 0.001 and 0.01 M (*p* < 0.001, ƞ^2^_p_ = 0.060; and *p* = 0.004, ƞ^2^_p_ = 0.039, respectively). We did not find any time x group interaction in the examined variables between SZ and BD groups.

#### 3.1.3. Differences in Niacin Responses between Bipolar Disorder and Healthy Controls

**Figure 4:**
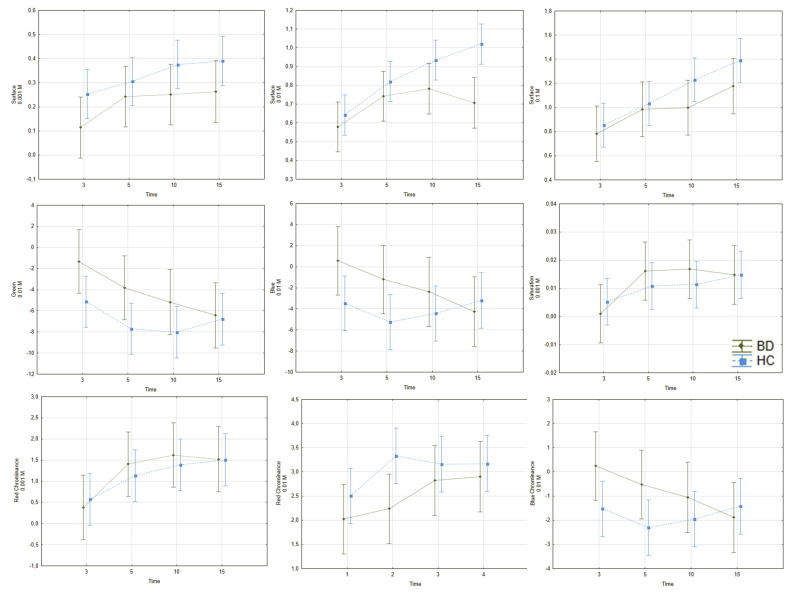
Differences in response to niacin solution between HC and BD. The blue line—HC group, the green line—BD group, the vertical axis—skin color, the horizontal axis—time of measurement in minutes. Diagrams represent group and within-subjects effects described in the Section 3.1.3. BD—bipolar disorder; HC—healthy control. (**a**) surface, 0.001 M; (**b**) surface, 0.01 M; (**c**) surface, 0.1 M; (**d**) green colour, 0.01 M; (**e**) blue colour, 0.01 M; (**f**) saturation, 0.001 M; (**g**) red chrominance, 0.001 M; (**h**) red chrominance, 0.01 M; (**i**) blue chrominance, 0.01 M.

As was shown in Figure 4 and Supplementary Table S1, we found differences in the surface effect at 0.001 and 0.01 M (*p* = 0.007, ƞ^2^_p_ = 0.026; *p* < 0.001, ƞ^2^_p_ = 0.041); green was observed at 0.01 M (*p* = 0.006, ƞ^2^_p_= 0.026) between HC and BD. There were time effects for the surface at 0.01 and 0.1 M (*p* < 0.001, ƞ^2^_p_ = 0.073; and *p* < 0.001, ƞ^2^_p_ = 0.068, respectively).

#### 3.1.4. The Frequency of Absent Response to AMN Solution across the Examined Group

An absent response to the AMN solution was defined as no changes in the color of flushing compared with a non-irritated fragment of skin. A lack of reaction was observed in 60.7% of the SZ group, 32.2% of the BD group and 28.9% of the HC group.

### 3.2. Associations between Sociodemographic Variables and Responses to AMN Solution

To grasp potential significant relations between the independent variables and dependent variables, such as gender, age and BMI, in the niacin-induced flushing results, correlations and Student’s *t*-test were computed.

We did not find any significant correlations between sociodemographic data (age, body mass, BMI, number of cigarettes smoked per day) and the niacin skin flush test in HC group (*p* > 0.05).

However, in the healthy men subgroup, more significant changes in green coloration, at 10 min after 0.001 M niacin solution application, was observed, as compared to healthy women (*p* < 0.05).

In BD group, there was a moderate negative correlation between duration of illness and green color (0.1 M) (*p* < 0.05, R = −0.41), blue color (0.1 M) (*p* < 0.05, R = −0.45) and blue chrominance (*p* < 0.05, R = −0.45), and a strong positive correlation between the number of cigarettes smoked per day and surface (0.1 M) (*p* < 0.05, R = 0.69). Women from the BD group had less remarkable red chrominance changes at 10 min after 0.01 M AMN application, compared to the men (*p* < 0.05).

In SZ group we found a weak positive relationship between BMI and green color (0.001 M) (*p* < 0.05, R = 0.27), blue color (0.001) (*p* < 0.05, R = 0.26) and blue chrominance (0.001 M) (*p* < 0.05, R = 0.26). Men from the SZ group had less significant changes in green coloration at 3 min after 0.001 M niacin solution application, as compared to the women (*p* < 0.05).

### 3.3. The Sensitivity and Specificity of SKINREMS

According to ROC curve results, the analysis revealed that chrominance at 3 min and 0.01 M solution had 71% sensitivity and 66% specificity, with regards to differentiating SZ from HC patients, while chrominance at 3 min and 0.001 M solution had 91% sensitivity and 72% specificity in differentiating SZ from BD patients. The variable with the highest predictive value, in reference to HC and BD groups, was green coloration at 5 min and 0.01 M (55% sensitivity and 54% specificity).

## 4. Discussion

The aim of our study was to create a new method for the objective assessment of niacin skin flush challenge results, and to verify the utility of the proposed tool in distinguishing individuals with schizophrenia and/or bipolar disorder from healthy subjects.

In 2010, Nadalin et al. published a review and proposed a procedure for conducting a subjective interpretation of the niacin test results [5]. According to the authors, a subjective assessment is the most frequently performed evaluation method. The first version of assessment of the niacin response was a 4-point scale, proposed by Ward et al., where 0 points was defined as a no erythema and 3 points was defined as erythema with edema beyond the area of the patch [26]. In 2001, the scale was redefined and divided into 7 points—1 point indicated no skin reaction, while 7 points meant an intense redness with a visible edema that starts to spread out, or edema bigger than the patch area. The scales allow us to estimate the value for each niacin concentration and the total niacin sum score [32].

In 2002, Puri et al. proposed the method of using a volumetric index, which was based on a 4-point scale, an erythema, the niacin concentration, and the time of the assessment after topical AMN application [33]. Despite modification and up-to-date techniques, the disadvantage of point rating scales is a high variability of scores, due to the subjectivity of a researcher carrying out the assessment.

In 2020, an article was published that also performed the subjective visual evaluation, rating the response to AMN by applying the scale rates from 0 to 3 [33]. As opposed to methods mentioned above, the SKINREMS is not based on the subjective scales, but is calculated automatically by raw values, and it allows for more precise and objective assessment of intensity of reaction, including not only red color but also components imperceptible to the human eye [34].

There is also a method based on the observation that prostaglandins produced during AMN stimulation lead to capillary vasodilation and an increase in blood flow, and these processes could be easily measured by optical reflection spectroscopy (ORS) [10]. The spectroscopy was also used to evaluate the methods based on the 7-point scale.

The ORS enables one to objectively assess the color changes by using a double peak (542 and 577 nm) of oxyhemoglobin absorption, by taking into account the differences in individuals’ basic skin color (by estimating the difference between reflection intensities before and after stimulation of AMN) [10].

Nevertheless, Smesny et al. noticed that spectroscopy assessment allows the disclosure of group differences only in lower niacin concentrations, and with regard to higher AMN concentration, visual assessment is more accurate. This dissimilarity is caused by the fact that both methods put weight on two examined parameters to a different degree. In the higher concentration, strong erythema rapidly merges into edema, which could not reveal skin redness. The ORS measure only assesses skin redness, so would not have been able to assess these differences [10].

Another advanced method offering a high degree of objectivity was proposed by Gorniak and Rybakowski [35]. The researchers carried out their examination in a dark room using artificial light. The color was coded as an intensity combination of three colors. The obtained photos were assessed using a graphic program and the analysis was based on the difference in color value between two skin areas—exposed and non-exposed to the AMN [35]. The implementation of the method proposed by Polish researchers requires special conditions, which are often difficult to achieve in clinical practice (i.e., an incandescent lamp, a dark room, an advanced graphic program). The method is similar to that proposed by us. However, the SKINREMS can be performed without such stringent conditions.

In 2003, Messamore et al. proposed the laser Doppler flowmetry method (LDF), which measures microcirculation in a non-invasive way. The recorded results are aggregated and transferred to the computer, which allows objective analysis [22]. One of the LDF’s disadvantages is patient-related parameters; a movement during the examination may be an important factor affecting the measurement. What is more, a severe reaction during the niacin performance could lead to dermal edema, capillary compression, and as a result, an improper assessment [36].

To date, for obtaining high accuracy in niacin skin challenge assessment, an examination must be performed, which is usually time-consuming and convoluted given the clinical practice methods. The methods proposed above seem to be favorable in scientific research, however, they go beyond the framework and time possibilities of clinical practice.

As described by us, the assessment performed by medical personnel requires taking photos and marking the areas and scale in the computer program. It is worth mentioning that after preliminary data analysis, the MATLAB algorithm can be modified and adapted to the examined groups (for example, factors related to illness or lifestyle behaviors). Unlike visual methods, the use of an automatic processing algorithm reduces the uncertainty of results caused by random errors.

Numerous studies have reported an absence or diminishing of responses to AMN to various degrees among schizophrenic individuals. In our study, the absent response was detected in 60.7% of individuals suffering from schizophrenia, and the proposed method could predict schizophrenia with 71% sensitivity and 66% of specificity.

The evaluation based on the visual method indicated an absent or diminished reaction to AMN in 83% [26], 90% [37] and 49.2% [38] of patients with schizophrenia. The discordant results could be an effect of heterogenous methodology, sample size dissimilarity, or population traits and their characteristics (a different stage or course of the illness, or factors involved in the pathophysiology of the illness). In studies where the assessment was based on visual inspection, results could be highly biased, due to the lack of participants’ diagnoses blinding color perception during the assessment of the photographed site.

In the study based on visual assessment, Ward et al. calculated a sensitivity of 83% and specificity of 77% for the reaction to the 0.01 M solution of AMN after 5 min of exposure [26]. In the study by Puri et al., the niacin skin test had a sensitivity of 90% and a specificity of 75%, at a concentration of 0.001 M at the 15-min time-point [37]. The study by Liu et al. showed that the most suitable parameter for distinguishing schizophrenic patients was the assessment done at 10 min after application at 0.001 niacin concentration, with 49.2% sensitivity and 92.5% specificity of the test [38].

Using a volumetric index, Puri et al. calculated a sensitivity of 78% and a specificity of 65% [33]. Smesny et al. compared two methods’ sensitivity and specificity (a visual scale and spectroscopy). The most prominent variable turned out to be a response after 6 min at 0.001 M concentration of niacin (72% sensitivity, 88% specificity). However, the analysis of data from the visual inspection showed that the most powerful discrimination was possible at 0.01 and 0.001 M concentration, after 11 min of the performed test—sensitivity was at 84% and specificity at 76%. What is more, the authors showed that the most useful parameter for distinguishing schizophrenic patients from healthy persons was the combination of variables—min/step value—which including both time and concentration at 6 min of 0.001 M, and at 21 min of 0.01 M—sensitivity was 92% and specificity 84% [10]. According to the Portland study, the blood flowmetry method indicated niacin response abnormality with a sensitivity of 32% and a specificity of 95%, for differentiating schizophrenia and healthy controls, and 32% and 87% respectively for differentiating schizophrenia and bipolar disorder patients [11]. Yao et al. pointed out that the niacin response abnormality predicted schizophrenia with 31% sensitivity and 95% specificity, compared to the healthy controls, and with 31% sensitivity and 97% specificity compared to the bipolar disorder individuals [11].

A possible common pathophysiological mechanism underlying both schizophrenia and bipolar disorder should be considered as a factor that makes it difficult to differentiate between these disorders [31]. Therefore, achieving higher sensitivity and specificity is probably unobtainable [39,40].

The etiopathogenesis of schizophrenia is multifactorial, without a single underlying cause [1]. The possibility of several various pathomechanisms creates the need to categorize schizophrenia. The endophenotype concept could help to identify subtypes for precision psychiatry [8].

Taking into account the studies mentioned above, the niacin skin response seems to be the most replicable, and makes it easy to measure a clinical manifestation of impaired phospholipid metabolism and the activation of inflammatory processes in individuals with schizophrenia. What is more, lastly, studies have shown that an impaired response to niacin is linked with the severity of symptoms and transition to psychosis in the ultra-high risk for psychosis group (UHR) [41]. The proposed tool enables an easy, quick and cheap way to assess the absent or impaired reaction to niacin in everyday clinical practice. However, there are still insufficient therapeutic proposals based on the abnormal responses in the niacin challenge. Supplementation of essential fatty acids (EFAs) is suggested. More studies are needed to clearly determine the clinical approach [41].

In practical terms, the development of a simple tool, available to any physician, would also allow one to quickly expand the databases with homogeneous methodological results, and in the future, translate them into practice through individualized treatment, based on the assessment of the biochemical background.

## 5. Strengths and Limitations of the Study

The study has some strengths and limitations. We created the new method, which is more objective and complex in determining niacin skin flush test results. The blinding of the participants’ diagnosis for the researcher performing the test makes it more reliable. Including confounding factors, related to lifestyle and the clinical picture of patients, which could affect results of the niacin skin flush test, is another advantage.

Despite the fact that our study seems to broaden the actual knowledge regarding niacin response in selected neuropsychiatric conditions, it has some limitations which should be addressed. We assessed the patient groups only with corresponding symptom severity scales. Using the interview guide for making the major DSM-5 diagnoses—SCID-5—in all study groups should strengthen the diagnostic accuracy and reliability.

Another limitation of this study is the relatively small study group, not entirely comparable regarding age and gender. This potentially could have some confounding effect on the obtained results regarding group differences in niacin response, however, we have undertaken an attempt to control for such hypothetical impact, by analyzing whenever there were any correlations between selected sociodemographic characteristics and niacin response-variables, which proved to significantly differ between the groups, according to MANOVA results. Additionally, to control for the impact of gender, in every group separately, we have compared mentioned niacin-related variables between males and females. Nevertheless, future studies including more than two research groups should recruit participants more equated in terms of age, gender, BMI and smoking.

## 6. Conclusions

1. Skin Reaction Measurement Computer System (SKINREMS) is a useful method for quick and objective evaluation of responses to the niacin skin flush test, and initial differentiation of individuals with schizophrenia, bipolar disorder and healthy persons.
2. The highest predictive value was characterized by red chrominance, at 3 min after skin challenge in 0.01 AMN concentration, for schizophrenia and healthy controls, green color at 5 min after niacin skin challenge at 0.01 M AMN concentration, for bipolar disorder and healthy controls, and red chrominance at 3 min after niacin skin challenge at 0.001 M AMN concentration, for bipolar disorder and schizophrenia.
3. The sensitivity and specificity of the SKINREMS were 71% and 66%, respectively, for compared schizophrenia patients and healthy individuals; 55% and 54%, respectively, for compared bipolar patients and the healthy individuals; and 91% and 72%, respectively, for compared schizophrenia and bipolar patients.
4. Schizophrenia and bipolar disorder are conditions with a multifactorial pathogenesis. This fact could explain why the results of studies are inconsistent and the niacin skin flush test could not detect the disease in all affected individuals. Nevertheless, the niacin skin flush test may be a good tool for revealing specific traits (abnormal responses to AMN) that are a manifestation of impaired lipids metabolism.
5. This method could provide a basis for stratifying patients for further research and selecting the most suitable therapy.
6. The focus on abnormal biochemical reactions and disruptions in inflammatory processes that occur in some groups of psychiatric patients, together with therapy based on pathophysiological markers, may provide a promising approach in the management of mental disorders.

## Figures and Tables

**Table 1:** Characteristics of the examined population.

Examined Factor	SZ Group	BD Group	HC Group	Results
Age * (years)	34	42	27	H = 13.03; *p* = 0.0015 BD > HC; SZ > HC
(Me)				
Gender	27 (48)	7 (24)	27 (60)	χ^2^ = 9.17; *p* = 0.0102 HC > BD; SZ > BD
*n* (% male)				
BMI (kg/m^2^) *	26.9	27.5	23.2	H = 10.05; *p* = 0.066; BD > HC
(Me)				
Smokers	21 (37.5)	11 (37.9)	8 (17.8)	χ2 = 4.74; *p* = 0.0295 SZ > HC
*n* (%) *				
Number of cigarettes	15	20	10	NS
per day				
(Me)				
Duration of illness (months)	96	84	NA	NS
(Me)				
Number of hospitalizations	2	3		NS
(Me)				
Atypical antipsychotics used	50 (89.29)	9 (31.03)		χ^2^ = 30.53; *p* < 0.001
*n* (%)				
Equivalents of olanzapine (mg)	15	15		NS
(Me)				
PANSS	56	NA		NA
(Me)				
YMRS	NA	7.5		NA
(Me)				
MADRS	NA	17		NA
(Me)				

* *p* < 0.05; Me—median; H—Kruskal–Wallis non parametric ANOVA test; Z—Z-score for U Mann–Whitney Test; χ^2^—chi-square statistic; NS—not significant; NA—not applicable; PANSS—Positive and Negative Syndrome Scale; YMRS—Young Mania Rating Scale; MADRS—Montgomery–Asberg Depression Rating Scale, SZ—schizophrenia; BD—bipolar disorder; HC—healthy control.

## Supplementary Materials

The following are available online at https://www.mdpi.com/2077-0383/9/6/1848/s1, Table S1: Differences in response to niacin solution between examined groups.
