# Supplementary material for: SKINREMS—A New Method for Assessment of the Niacin Skin Flush Test Response in Schizophrenia
Source: J Clin Med. 2020 Jun 13;9(6):1848. doi: 10.3390/jcm9061848 (PMC7356909; doi:10.3390/jcm9061848)
Supplement: Supplementary file 1 [file jcm-09-01848-s001.pdf]

Table S1. Differences in response to niacin solution between examined groups

Schizophrenia vs Healthy Control				
**Component**	**Concentration of AMN**	*Statistic values*		
		Time effect	*Group effect*	*Time x Group effect*
**Surface**	0.001M	**F=5.4567, *p*=0.001, η^2_P=0.034**	**F=23.44, *p*<0.001, η^2_P=0.056**	NS
	0.01M	**F=18.62, *p*<0.001, η^2_P=0.124**	**F=25.32, *p*<0.001, η^2_P=0.060**	NS
	0.1M	**F=9.33, *p*<0.001, η^2_P=0.066**	**F=12.49 , *p*<0.001, η^2_P=0.031**	NS
**Red colour**	0.001M	NS	NS	NS
	0.01M	NS	NS	NS
	0.1M	NS	NS	NS
**Green colour**	0.001M	**F=4.11, *p*=0.007, η^2_P=0.030**	NS	NS
	0.01M	F=3.59, *p*=0.014	**F=8.92, *p*=0.003, η^2_P=0.022**	NS
	0.1M	NS	F=4.03, *p*=0.046	NS
**Blue colour**	0.001M	NS	NS	NS
	0.01M	NS	F=5.79, *p*=0.017	NS
	0.1M	NS	NS	NS
**Hue**	0.001M	F=3.67, *p*=0.012	NS	NS
	0.01M	NS	NS	NS
	0.1M	NS	NS	NS
**Saturation**	0.001M	F=3.63, *p*=0.013	NS	NS
	0.01M	NS	**F=8.60, *p*=0.004, η^2_P=0.021**	NS
	0.1M	NS	NS	NS
**Value**	0.001M	NS	NS	NS
	0.01M	NS	NS	NS

	0.1M	NS	NS	NS
**Red chrominance**	0.001M	**F=6.08, *p*<0.001, η^2_p=0.044**	**F=8.60, *p*=0.004, η^2_p=0.021**	NS
	0.01M	**F=6.33 , *p*<0.001, η^2_p=0.046**	**F=17.40, *p*<0.001, η^2_p=0.042**	NS
	0.1M	NS	F=5.10, *p*=0.025	NS
**Blue chrominance**	0.001M	NS	NS	NS
	0.01M	NS	F=5.94, *p*=0.015	NS
	0.1M	NS	NS	NS
Bipolar Disorder vs Healthy Control				
**Component**	**Concentration of AMN**	*Statistic values*		
		Time effect	*Group effect*	*Time x group effect*
**Surface**	0.001M	NS	**F=7.50, *p*=0.007, η^2_p=0.026**	NS
	0.01M	**F=7.46, *p*<0.001, η^2_p=0.073**	**F=12.18, *p*<0,001, η^2_p=0.041**	NS
	0.1M	**F=6.89, *p*<0.001, η^2_p=0.068**	NS	NS
**Red colour**	0.001 M	NS	NS	NS
	0.01 M	NS	NS	NS
	0.1 M	NS	NS	NS
**Green colour**	0.001M	NS	NS	NS
	0.01M	NS	**F=7.53, *p*=0.006, η^2_p=0.026**	NS
	0.1 M	NS	NS	NS
**Blue colour**	0.001M	NS	NS	NS
	0.01M	NS	F=4.58, *p*=0.033	NS
	0.1M	NS	NS	NS
**Hue**	0.001M	NS	NS	NS
	0.01M	NS	NS	NS

	0.1M	NS	NS	NS
**Saturation**	0.001M	F=2.70, *p*=0.046	NS	NS
	0.01M	NS	NS	NS
	0.1M	NS	NS	NS
**Value**	0.001M	NS	NS	NS
	0.01M	NS	NS	NS
	0.1M	NS	NS	NS
**Red chrominance**	0.001M	F=3.88, *p*=0.010	NS	NS
	0.01M	NS	F=5.42, *p*=0.021	NS
	0.1M	NS	NS	NS
**Blue chrominance**	0.001M	NS	NS	NS
	0.01M	NS	F=4.57, *p*=0.033	NS
	0.1M	NS	NS	NS
Schizophrenia vs Bipolar Disorder				
**Component**	**Concentration of AMN**	*Statistic values*		
		Time effect	*Group effect*	*Time x group effect*
**Surface**	0.001M	**F=6.23, *p*<0.001, η^2_p=0.054**	NS	NS
	0.01M	**F=6.72, *p*<0.001, η^2_p=0.058**	NS	NS
	0.1M	**F=5.16, *p*=0.002, η^2_p=0.045**	NS	NS
**Red colour**	0.001M	NS	NS	NS
	0.01M	NS	NS	NS
	0.1M	NS	NS	NS
**Green colour**	0.001M	NS	F=5.24, *p*=0.023	NS
	0.01M	F=3.77, *p*=0.011	NS	NS
	0.1M	NS	NS	NS
**Blue colour**	0.001M	NS	NS	NS

	0.01M	NS	NS	NS
	0.1M	NS	NS	NS
**Hue**	0.001M	NS	NS	NS
	0.01M	NS	NS	NS
	0.1M	NS	NS	NS
**Saturation**	0.001M	**F=5.09, *p*=0.002, η^2_P=0.044**	NS	NS
	0.01M	NS	NS	NS
	0.1M	NS	NS	NS
**Value**	0.001M	NS	NS	NS
	0.01M	NS	NS	NS
	0.1M	NS	NS	NS
**Red chrominance**	0.001M	**F=7.03, *p*<0.001, η^2_P=0.060**	**F=9.68, *p*=0.002, η^2_P=0.029**	NS
	0.01M	**F=4.50, *p*=0.004, η^2_P=0.039**	NS	NS
	0.1M	NS	NS	NS
**Blue chrominance**	0.001M	NS	NS	NS
	0.01M	NS	NS	NS
	0.1M	NS	NS	NS

AMN - aqueous methyl nicotinate; η^2_P - partial eta squared
